# Cytotoxicity, Genotoxicity and Disturbance of Cell Cycle in HepG2 Cells Exposed to OTA and BEA: Single and Combined Actions

**DOI:** 10.3390/toxins11060341

**Published:** 2019-06-14

**Authors:** Ana Juan-García, Josefa Tolosa, Cristina Juan, María-José Ruiz

**Affiliations:** 1 Laboratory of Food Chemistry and Toxicology, Faculty of Pharmacy, University of Valencia, Av. Vicent Andrés Estellés s/n, Burjassot, 46100 València, Spain; josefa.tolosa@uv.es (J.T.); cristina.juan@uv.es (C.J.); m.jose.ruiz@uv.es (M.-J.R.); 2 ProtoQSAR, CEEI, Avda. Benjamin Franklin 12, Paterna, 46980 Valencia, Spain

**Keywords:** ochratoxin A, beauvericin, mixtures, HepG2 cells, genotoxicity, cell cycle

## Abstract

Mycotoxins are produced by a number of fungal genera spp., for example, *Aspergillus*, *Penicillium*, *Alternaria*, *Fusarium*, and *Claviceps*. Beauvericin (BEA) and Ochratoxin A (OTA) are present in various cereal crops and processed grains. This goal of this study was to determine their combination effect in HepG2 cells, presented for the first time. In this study, the type of interaction among BEA and OTA through an isobologram method, cell cycle disturbance by flow cytometry, and genotoxic potential by in vitro micronucleus (MN) assay following the TG 487 (OECD, 2016) of BEA and OTA individually and combined in HepG2 cells are presented. Cytotoxic concentration ranges studied by the MTT assay over 24, 48, and 72 h were from 0 to 25 µM for BEA and from 0 to 100 µM for OTA, while BEA + OTA combinations were at a 1:10 ratio from 3.4 to 27.5 µM. The toxicity observed for BEA was higher than for OTA at all times assayed; additive and synergistic effects were detected for their mixtures. Cell cycle arrest in the G0/G1 phase was detected for OTA and BEA + OTA treatments in HepG2 cells. Genotoxicity revealed significant effects for BEA, OTA, and in combinations underlining the importance of studying real exposure scenarios of chronic exposure to mycotoxins.

## 1. Introduction

The presence of mycotoxins in food and feed have associated toxicological effects in consumers such as nephrotoxicity, hepatotoxicity, teratogenicity, etc., or potential effects such as synergism, additive or antagonism when a combination of more than one mycotoxin occurs [1,2,3,4,5]. The overall evidence on mixture effects indicates that combined effects can arise when each mixture component is present at doses around or above its no-effect level and provides a strong basis for developing robust approaches to assess the risk of chemical mixtures to support decision making [6].

Studies of mixtures of mycotoxins are directed to find whether there is an interaction between them, and if so, whether this interaction potentiates or diminishes the toxic effects of mycotoxins tested individually. When there is no interaction of mycotoxins between them, their effect is described as additive; while if the combination increases or decreases, the effect expected is described as a synergistic or antagonistic interaction, respectively. For this purpose, several models can be used to study such interactions. The mathematical model “*Loewe Additivity*” uses the isobologram equation proposed by Chou and Talalay et al. [7,8], which involves plotting the dose–effect curves (defined to a certain inhibition level) for each compound and their combinations in multiple diluted concentrations as described elsewhere [1,2,3,9].

The presence and co-presence of more than one mycotoxin in food and feed due to the ability of most *Fusarium* to simultaneously produce different mycotoxins is very common; thus, exposure to multi-mycotoxins often occurs [10,11,12]. Mixtures found worldwide have started to increase and become more diverse over the last decade due to the climatic changes and favorable growth conditions of different fungi spp. [12].

The EFSA has recently published a Draft Guidance document where a harmonized risk assessment methodology for combined exposure to multiple chemicals for all relevant areas is described (EFSA Journal 2018). There are specific requirements for chemical mixture risk assessment on the use of pesticides and food and feed additives [13,14], while for some mycotoxins, the sum of T-2 and HT-2 and the sum of aflatoxin B1 (AFB1), aflatoxin B2 (AFB2), aflatoxin G1 (AFG1), and aflatoxin G2 (AFG2) are underpinned in Regulation (EC) 1881/2006 [15]; however, neither beauvericin (BEA) nor ochratoxin A (OTA) are included.

For detecting genotoxicity, micronuclei (MN) induction assay has been accepted, validated, and recently updated in the Test Guideline 487 (TG 487) by the OECD [16]; and the inclusion of flow cytometry in the new TG 487 is a novelty which allows to determine cell cycle effect and MN-induction simultaneously [17,18]. Most of the articles published perform in vitro detection of MN through cytokinesis-block micronucleus (CBMN) assay for genotoxicity studies of mycotoxins produced by different fungal genera (*Fusarium*, *Penicillium*, and *Aspergillus*). Studies for Ochratoxin A (OTA), citrinin (CTN), patulin (PAT), beauvericin (BEA) [19,20,21,22,23,24,25,26], and aflatoxin B1 (AFB1) [27] can be found.

The new findings from cytotoxicity induced by binary mixtures of BEA + OTA in PK-15 cells and human leukocytes reveal that combined toxicity is higher than predicted from individuals; in fact, synergism and additive effects have been reported [28].

The in vitro system HepG2 cells are commonly used in toxicological studies. Effects reported for BEA, OTA, and its combination are diverse and it depends on different factors such as intake dose, exposure frequency, and timing of functional assays; in fact, their combination effect in HepG2 cells is here presented for the first time. In this study, the type of interaction between BEA and OTA through an isobologram method was studied. It is also presented the results of studying cell cycle disturbance by flow cytometry and genotoxic potential by in vitro micronucleus (MN) assay following the TG 487 [16] for BEA and OTA individually and in different mixtures in HepG2 cells.

## 2. Results

### 2.1. Cytotoxicity of Individual and Combined Mycotoxins

**Figure 1:**
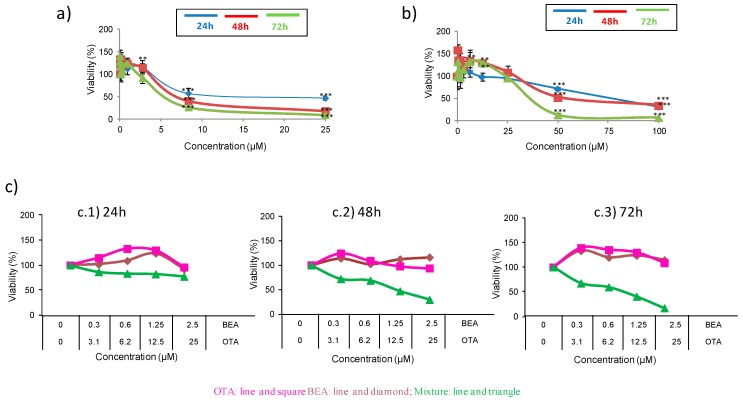
Cytotoxicity of BEA (**a**), OTA (**b**), and BEA + OTA (**c**) on HepG2 cells at mycotoxin exposures of 24, 48, and 72 h. The concentration for OTA mycotoxin was 0–100 µM (1:2 dilution), for BEA 0–25 µM (1:3 dilution), while for BEA + OTA at 1:10 ratio. * *p* ≤ 0.05.

Ceauvericin and OTA mycotoxins and their combination on HepG2 cells during 24, 48, and 72 h were studied through the MTT assay to evaluate the cytotoxicity. The assay was driven to determine the IC_50_ (inhibition of cell population to 50%) value. The IC_50_ values denoted that BEA was above OTA (BEA > OTA) in toxicity potential when individual treatment was evaluated (Figure 1a,b and Table 1); however, a dose-dependent manner (Figure 1c) of toxicity in IC_50_ values was achieved for mixtures of both mycotoxins. The highest toxic effect belonged to BEA at 72 h expressed by an IC_50_ level of 5.5 ± 0.071 µM. The IC_50_ values for each mycotoxin at different times of exposure are shown in Table 1.

For the BEA represented in Figure 1a, a concentration range of 2.5 to 25 µM viability values decreased in a time-dependent manner. At 24 h, reduction of viability was from 54 to 43%, whereas the reduction of viability was from 60 to 82% and from 90 to 73% for 48 h and 72 h, respectively. Figure 1b shows the concentration-dependent decrease of viability of HepG2 cells after OTA treatment. It produced a reduction of viability from 70 to 30% and from 65 to 47% at 24 and 48 h, respectively, whereas the reduction of viability for 72 h varied from 93 to 82%.

Figure 1c shows the concentration-dependent decrease in the HepG2 cell viability with combined treatment of BEA + OTA (1:10) at exposure times of 24, 48, and 72 h. The IC_50_ values were obtained for 24 and 48 h comprised in a concentration mixture range of BEA + OTA from 0.6 + 6.2 to 1.25 + 12.5 µM for both exposure times. The reduction of viability caused by the mycotoxin combination BEA + OTA (1:10) was different according to the exposure time: (i) at 24 h it oscillated between 6–49% and 3–26% for BEA and OTA, respectively, compared to the individual mycotoxin exposure; (ii) at 48 h, the reduction oscillated between 25–84% and 40–78% for BEA and OTA, respectively, compared to the individual mycotoxin exposure. Finally, at 72 h, reduction of viability oscillated between 19–77% and 31–83% for BEA and OTA, respectively, compared to the individual mycotoxin exposure.

**Figure 2:**
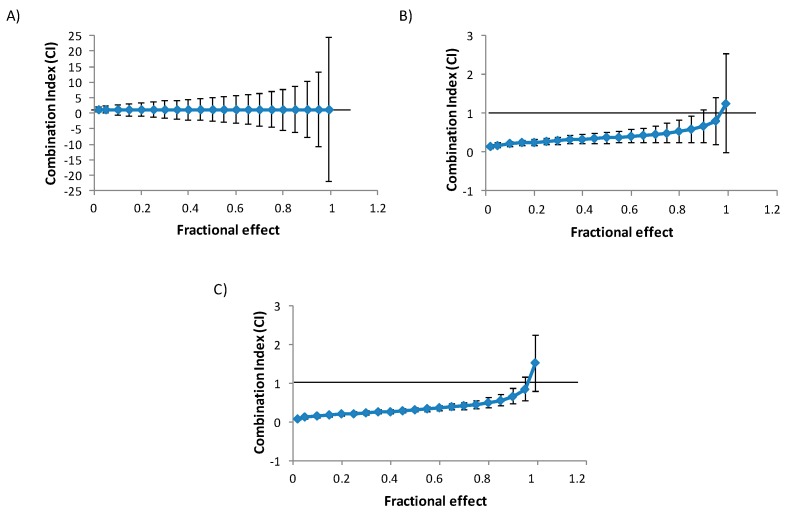
Combination index (CI) versus fractional effect curve as described by Chou and Talalay [7] model on HepG2 cells exposed to BEA + OTA (1:10) in binary combination. Each point represents the CI ± SD at a fractional effect as determined in our experiments. The line (CI = 1) indicates additivity, the area under this line synergism, and the area above the line antagonism. HepG2 cells were exposed during 24 (**A**), 48 (**B**), and 72 h (**C**) to BEA + OTA at a molar ratio of 1:10 (equimolar proportion).

The isobologram analysis was used to determine the type of interaction between BEA and OTA. The parameters *Dm*, *m*, and *r* of the binary and tertiary combinations, as well as mean combination index (CI) values are shown in Table 2. The CI versus fractional effect (*fa*) curves for BEA and OTA combinations in HepG2 cells are shown in Figure 2. Both Table 2 and Figure 2 demonstrated that the main effect caused by binary mixtures of BEA and OTA is synergism; however, for BEA + OTA mixture at 24 h, additive effect was observed as well as at 48 h for the highest CI evaluated (Figure 2 and Table 2).

### 2.2. Cell Cycle Distribution in Individual Mycotoxin Exposure

**Figure 3:**
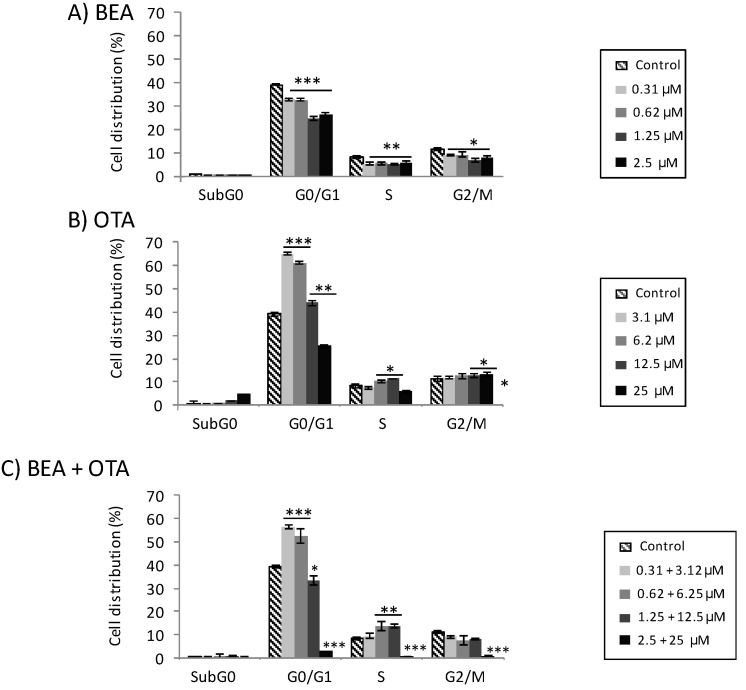
Cell cycle distribution in HepG2 cells exposed after 48 h to BEA (**A**), OTA (**B**), and BEA + OTA (1:10 molar proportion) (**C**). Data are expressed as mean ± SEM (*n* = 3). * p ≤ 0.05, ** *p* ≤ 0.01, and *** *p* ≤ 0.001 indicates significant differences compared to control.

Analysis of DNA content by flow cytometry provides a measure of cell cycle perturbation in HepG2 cells following exposure to BEA, OTA, and BEA + OTA (Figure 3).

Results for BEA exposure to all concentrations assayed resulted in statistically significant differences with respect to the control for all phases: G0/G1 (*p* ≤ 0.001), S (*p* ≤ 0.01), and G2/M (*p* ≤ 0.05) (Figure 3A). Effects observed correspond to a statistically significant decrease in the percentage of the number of cells compared to the control. For OTA exposure to all concentrations assayed, the results were a statistically significant increase with respect to the control for the G0/G1 phase (*p* ≤ 0.001 and *p* ≤ 0.01 for both higher and lower concentrations, respectively) (Figure 3B). Similarly, this happened for the S and G2/M phases for doses of 6.2 and 12.5 µM (S phase), and 12.5 and 25 µM (G2/M phase). The sub-G0 phase reported an increase of HepG2 cells at the highest concentration assayed (25 µM, *p* ≤ 0.01).

Regarding binary mixture BEA + OTA, a statistically significant increase was observed for the G0/G1 phase at concentrations of 0.31 + 3.12 and 0.62 + 6.25 µM and in the S phase for 1.25 + 12.5 and 2.5 + 25 µM compared to the control (Figure 3C).

### 2.3. Micronuclei Induction in Individual and Combined Mycotoxin Exposure

**Figure 4:**
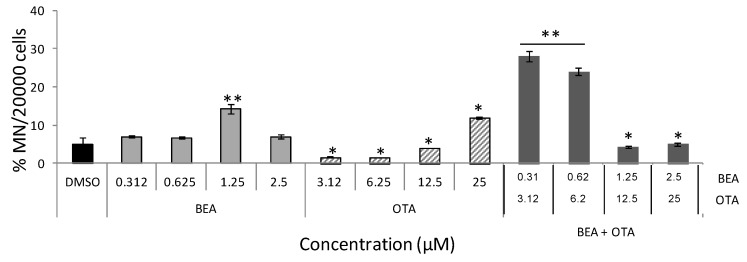
Induction of micronuclei in HepG2 cells treated by BEA, OTA, and BEA + OTA at several concentrations for 48 h. Results are expressed as a percentage of MN per 20,000 cells ± SEM (*n* = 3). *p* ≤ 0.05 (*) and *p* ≤ 0.001 (**), significantly different from the control.

Figure 4 collects MN frequencies in HepG2 cells exposed to BEA, OTA, and BEA + OTA. Among all two individual treatments, the increase effect on MN frequency was detected for BEA at a concentration of 1.25 μM (14.2 ± 1.1%, *p* ≤ 0.01). OTA revealed decreasing differences in respect to the no-treatment control for all concentrations except at 25 µM where statistically significant increases were detected (*p* ≤ 0.05). Regarding BEA + OTA combined treatments, increases in MN frequency at the lowest concentrations assayed were detected as follows: 28.3 ± 1.32% and 24.0 ± 0.97% for 0.31 + 3.12 and 0.62 + 6.25 µM (*p* ≤ 0.01), respectively.

## 3. Discussion

Cytotoxicity of BEA and OTA in HepG2 cells either in single or combined treatment was detected; subsequently, cell cycle alterations and micronuclei induction either individually or combined were assayed.

Among both mycotoxins, literature of OTA is wider than that compared to BEA; therefore, several studies performed in in vitro or in vivo models can be found. OTA has been classified as group 2B by the International Agency of Research in Cancer (IARC) and appears in the regulation EC No 1881/2006 [15] and recommendations [29] by the EC in respect to maximum levels in food and feed; in contrast, BEA is not classified as a carcinogen to humans and is not included in the Commission Regulation (EC) No 1881/2006 [15], which established maximum levels for specific contaminants to protect public health. However, efforts are focused in this mycotoxin, since in 2018 the EFSA published a Scientific Report related to in vivo toxicity and genotoxicity of BEA based on the fact that there are insufficient data to establish its reference values.

Regarding cytotoxicity, several authors studied OTA (0–200 µM at 4–24 h) in primary rat PT and LLC-PK1 cells [30], and IC_50_ values were of 1.1-fold higher and 0.3-fold lower, respectively, to each cell line compared to those obtained for HepG2 cells at 24 h in this study. In BME-UV1 and MDCK cells, OTA IC_50_ values were 0.8 and 1 µg/mL, respectively, showing a high sensitivity in this cell type compared with other cell models [31]. The HepG2 cells exposed to OTA (10–50 µM) reached an IC_50_ value 0.6-fold lower than that obtained in this study for the same period of time (24 h). This can be related with the type of cells used; in this study, HepG2 were used, which are characterized by containing high enzymatic activity. Several studies have revealed alterations in different enzyme activities after OTA exposure, indicating such effect as a potential target for OTA mycotoxin [32].

On the other hand, cytotoxic studies of BEA can also be found. CCRF-CEM cells exposed to BEA (1–10 µM) for 24 h [33] revealed an IC_50_ value 9.8-fold smaller than that of the HepG2 cells in this study. In HT-29 cells, IC_50_ values were closer to those reported here differing 0.8- and 0.7-fold lower for 24 and 48 h, respectively; while for Caco-2 cells, it was 0.6- and 0.5-fold lower for 24 and 48 h, respectively [34,35]. In general terms, comparing data from the literature with the results of this work, BEA IC_50_ values of HepG2 cells were smaller than those obtained with other cell lines; however, for the cell line CHO-K1, higher cytotoxicity was revealed since IC_50_ values were lower than those obtained in this study (1.2- and 3.18-fold lower for 24 and 72 h, respectively) [36]. An explanation for HepG2 sensitivity might be related to the high enzyme activity of these cells and the possibility that BEA exerts cytotoxicity associated to its hexadepsipeptide structure which is able to affect DNA migration, intracellular calcium levels, and apoptosis, as reported [28].

A study on PK-15 cells at fixed doses of BEA and OTA revealed decreases in viability for BEA at 35% (5 µg/mL, 24 h) and 26% (0.5 µg/mL, 48 h); while for OTA at 32% (5 µg/mL, 24 h) and 23% (0.5 µg/mL, 48 h) but no IC_50_ values were reached for any mycotoxin [37]. However, human leukocytes exposed to BEA, OTA, and BEA + OTA after 24 h, reached IC_50_ values 2.5- and 4.74-fold lower for BEA and OTA, respectively, compared to those for HepG2 cells reported in this study [28], which coincides with our results of OTA being less toxic than BEA. When studying BEA + OTA (at a 1:10 ratio) (24 h) additive and synergism effects were observed in PK-15 cells [28]; at short times (24 h) in our study, only the additive effect was observed (see Table 2 and Figure 2). Differences in such combination effects could be associated to cell type but also to the concentration assayed. Klaric et al. [28] assayed from 3- to 5-times lower than the concentration of BEA and OTA, respectively, when combined, compared to the ones presented here where the effect was measured with the isobologram analysis, while Klaric et al. [28] reported effects comparing prediction between expected and measured values.

Cytotoxicity observed in HepG2 cells after exposure to mycotoxins BEA and OTA can interfere in cell proliferation; so, the study of phases in cell cycle distribution (Figure 3) and its association with MN induction were studied (Figure 4). In general terms, cell cycle accumulation in the G0/G1 phase was detected for OTA (all concentrations except at 25 µM) and its combination with BEA (at the lowest concentrations assayed), while for BEA a decrease in G0/G1 was detected, revealing induction of cell death at the concentrations assayed. The HepG2 cells need 48 h to occur 1.5–2 times more than the normal cell cycle division, which is the condition needed to perform the MN assay according to OCDE TG 487. Cell cycle and MN assays were performed in the same conditions to associate alterations in cell cycle and MN induction as described by Juan-García et al. [18]. Among that, although there are few studies carried out in evaluating genotoxicity for BEA and OTA, to our knowledge, this is the first report on BEA, OTA, and BEA + OTA genotoxicity determined by flow cytometry, as approved in the reviewed version of OCDE TG 487 from July 2016.

Results for BEA in the cell cycle disturbed the distribution of phases compared to the control as described in Section 2.2; however, BEA 1.25 µM reported differences in all cell cycle phases in respect to the control (Figure 3A) and the strongest MN induction (Figure 4). Positive results have been reported for genotoxicity of BEA through cytokinesis-block micronucleus (CBMN) assay [26] and the COMET assay [28] as well as negatives [38]. Our results coincide with those positives which for all concentrations BEA MN-induction (here through flow cytometry) in HepG2 cells was observed but it was only statistically significant at 1.25 µM.

For OTA, a marked arrest in the G0/G1 phase at the lowest concentration assayed was detected (Figure 3B), which reveals that the HepG2 cells had everything in the cells ready for DNA synthesis but no DNA division happened. This point is associated with the fact that no increase in MN induction was detected unless OTA-high doses were tested (Figure 4), coinciding with other authors [20]. Other authors have compared genotoxic assays (COMET and CBMN) in HepG2 cells exposed to OTA revealing formation of MN and associating this at least partly to clastogenic effects; although no additional assay to contrast the results were performed [19]. In the present study, MN formation was measured simultaneously with cell cycle and the results were linked (Figure 3 and Figure 4). Previously, we performed this procedure for DON, 3-ADON and 15-ADON in the same cell line, and arrest in the G0/G1 phase was associated with MN induction and the procedure followed was the same as here (OECD TG487 reviewed in 2016 for cytoplasma MN detection in interphase cells) [18]. Several cell models, experimental conditions, and procedures have been used to study OTA-MN induction [21,39]. A recent study has been published using in Vero cells where OTA (25 μM, 30 min) produced a significant increase in the MN total number through CBMN assay [20]. It is important to mention that OTA is known as a DNA adducts-inductor [20]. Those chromosome breaks may point to a clastogenic mode of action if unrepaired. Thus, it is important to refer to the basis of each method and procedure followed; since MN generated through clastogenic or aneugenic events, are irreversible and persistent, and time that approximates a 1.5–2 normal cell cycle of the cell line is crucial.

Finally, the mycotoxin mixture OTA + BEA in HepG2 cells showed an arrest in the G0/G1 and S phases (Figure 3C) correlated with the MN induction detected (Figure 4). Mixture tested resulted in a stable solution (see Section 4.3). The natural occurrence of both mycotoxins has been reported in many maize fields and maize-based products worldwide [9,40]. According to the literature, BEA is not an inducer of the deletion of a DNA excision repair system gene [38], while oppositely, OTA has been reported as a DNA adducts-inductor [20]; therefore, this could support our results. DNA strand breaks through a COMET assay have been detected using a different in vitro system (PK-15 cells and human leukocytes) and exposure time for this combination and at doses lower than those presented in here (BEA: 0.1 and 0.5 μM; OTA: 1 and 5 μM). Coinciding with our results, a positive genotoxic effect was obtained.

In conclusion, individual and combined BEA and OTA cytotoxicity in HepG2 cells was studied. In binary mixtures, synergism and additive effect prevailed. Both OTA and BEA and their combinations provoked disturbance in the cell cycle and affected MN induction, thus underlining the importance of studying real exposure scenarios of chronic exposure to these toxins.

## 4. Materials and Methods

### 4.1. Reagents

All reagents and cell culture components were standard laboratory grade from Sigma–Aldrich (St. Louis, MO, USA). The standard of OTA (MW: 403.815 g/mol) and BEA (MW: 783.95 g/mol) was purchased from Sigma–Aldrich (St. Louis, MO, USA). Methanol (MeOH) was obtained from Fisher Scientific (Madrid, Spain). For MTT assay, thiazolyl blue tetrazolium bromide (approx. 98%; M2128-10G) was purchased from Sigma–Aldrich. A Milli-Q water purification system (Millipore, Bedford, MA, USA) permitted the obtainment of the deionized water (<18 MΩ cm resistivity). Stock solutions of mycotoxins were prepared in MeOH (OTA and BEA) and maintained at −20 °C in the dark. The final concentration of MeOH in the medium was ≤1% (*v*/*v*) as per established for performing this in vitro assays.

### 4.2. Cell Culture

Growth of HepG2 cells (ATCC HB-8065) was possible using Dulbecco’s Modified Eagle’s Medium (DMEM, Sigma–Aldrich) supplemented with antibiotic-free 10% newborn calf serum (NCS; Invitrogen, Christchurch, New Zealand), 100 U/mL penicillin, and 100 mg/mL streptomycin (Sigma–Aldrich). Cells were used between passages 12 and 19. The cells were trypsinized (Trypsin-EDTA, Sigma–Aldrich) and resuspended in complete medium in a 1:3 split ratio to perform the sub-cultivation. The procedure was repeated once or twice a week according to the monolayer confluence in flasks with filter screw caps (TPP, Trasadingen, Switzerland) at 37 °C in a humidified atmosphere and 5% CO_2_.

### 4.3. Mycotoxin Exposure

In this study, concentration of mycotoxins and exposure time were two important factors to consider. Twenty-four, 48, and 72 h were the exposure times assayed in HepG2 cells for BEA and OTA either individually and combined. The situation of 72 h was considered due to the accumulation process possibly occurring because of the physicochemical properties of these compounds. Individual treatment was assayed at a concentration range of 0 to 25 µM for BEA (1:3 dilution), and at a range of 0 to 100 µM for OTA (1:2 dilution). For combination mixtures, individual treatment data were crucial for selecting starting concentration. Nevertheless, parallel assays of individual and combinations were performed for exact evaluation of combinatory effects. Concentration for combinations of both mycotoxins at a dilution ratio 1:10 (BEA + OTA) was from 3.3 to 27.5 µM, including four dilutions of each mycotoxin combination for BEA (0.3, 0.6, 1.25, and 2.5 µM) and OTA (3.1, 6.2, 12.5, and 25 µM).

### 4.4. In Vitro Cytotoxicity

The protocol published by Ruiz et al. [41], with slight modification, was followed to evaluate the cytotoxicity. It describes the MTT assay which consists of measuring the viability of cells by determining the reduction of the yellow, soluble tetrazolium salt only in cells that are metabolically active via a mitochondrial reaction to an insoluble, purple formazan crystal. The concentration of cells per well was 2 × 10^4^ cells/well for culture plates of 96-wells. Cells adhered to the plates after 18–24 h, which was the elapsed time before proceeding with mycotoxin additions. Section 4.3 details the dilutions and combinations of mycotoxins tested for BEA and OTA. Fresh supplemented medium was used for preparing serial dilutions in the designed plate, while controls were prepared with culture medium with <1% methanol. After passing 24, 48 or 72 h, the medium containing the mycotoxin was removed and 200 µL of fresh medium containing 50 µL of MTT solution was added (5 mg/mL; MTT powder dissolved in phosphate buffered saline). Plates were kept for 4 h at 37 °C in darkness and afterwards the MTT was removed and 200 µL of DMSO and 25 µL of Soerensen’s solution (composition: glycine 0.4 µM + 0.1 µM NaCl, pH 10.5) was added to each well. Plates were brought to read at 570 nm with the ELISA plate reader Multiskan EX (Thermo Scientific, Waltham, MA, USA). Replicates consisted of each mycotoxin combination plus a control tested in three independent experiments. Mean inhibition concentration (IC_50_) values were calculated from full dose–response curves using the four parameter logistic equation with the SigmaPlot program. Three independent experiments were performed with eight replicates each.

### 4.5. Experimental Design and Combination Index

The isobologram analysis (Chou-Talalay model) was used to determine the type of interaction (synergism or antagonism effect) that occurs when mycotoxins studied were in combination. Chou-Talalay’s model allows characterizing the interactions induced by combinations of mycotoxins, but no mechanisms associated can be elucidated. Combination effects are possible to analyze by the median-effect/combination index (CI)-isobologram equation by Chou [42] and Chou and Talalay [7]. The model involves plotting the dose–effect curves for each mycotoxin and its combinations in multiple diluted concentrations. There are several parameters included in the equation as *Dm* (the median-effect dose), *fa* (fraction affected by concentration), and *m* (coefficient signifying the shape of the dose–effect relationship) [7]. Therefore, both the potency (*Dm*) and shape (*m*) parameters are crucial for the evaluation.

The term combination index (CI) is included in Chou and Talalay’s [42] method. According to the CI synergism, additive, and antagonism are, respectively, correlated as follows: CI < 1, =1, and >1. CalcuSyn software version 2.1. (Biosoft, Cambridge, UK, 1996–2007) was used to study the types of interactions assessed by isobologram analysis. It was decided to report CI_25_, CI_50_, CI_75_, and CI_90_ doses to produce toxicity at 25%, 50%, 75%, and 90%, respectively.

### 4.6. Cell Cycle Analysis by Flow Cytometry

Vindelov’s PI staining solution previously described [43] was used. PI is a DNA intercalating agent that only stains stoichiometrically the DNA of cells in the late phases of cell death, when the integrity of both cellular and nuclear membranes is lost. Cell proliferation and cell cycle distribution was performed using BD FACSCanto™ Flow Cytometer (Beckton–Dickinson, Italy) with FACSDiva software version 6.1.3 (BD Biosciences, San José, CA, USA, 2007).

For this assay, plates of 6 wells were used and the concentration of cells was 4.8 × 10^5^. Cells were seeded for 24 h and exposed for 48 h to BEA at 0.31, 0.62, 1.25, and 2.5 μM for 48 h; and OTA at 3.12, 6.25, 12.5, and 25 μM for individual treatment. For combinations, range doses went from 3.43 to 27.5 µM including four dilutions of each combination. Then, cells were trypsinized and incubated at 37 °C for 30 min with 860 μL of fresh medium containing 29 ng/mL of Vindelov’s PI staining solution. Cell cycle analysis was carried out as described by Minervini et al. [44], by rectangular fitting (CYLCHRED software, Beckton–Dickinson, Milan, Italy) using 1024 channels, which produced histograms with a single G0/G1 peak at channel 200 when DNA was diploid, an S-peak between channels 200 and 400 when DNA was replicating, a G2/M peak at channels 400 when DNA was tetraploid, and a Sub-G0 peak (debris peak), between 100 and 200 when DNA was hypodiploid or damaged. The reduced coefficient of variation (CV) obtained in this study was the result of the high resolution reached by proper alignment. The positive control used was cicloheximide (CLX) (40 μg/mL), known as synthesis of proteins inhibitor that leads to cellular quiescence and cell death by apoptosis. Three independent experiments were performed for OTA and BEA and at least 10,000 cells were analyzed for each sample.

### 4.7. Genotoxic Evaluation by Micronucleus Detection through Flow Cytometry

Litron In Vitro Microflow Kit (Litron Laboratories, Rochester, NY, USA) was used for the micronucleus assay on HepG2 cells exposed to mycotoxins by flow cytometry following the OECD TG487. Conditions set for this assay point that it must be carried out after exposure of cells to the toxic during a time that approximates a 1.5–2-times normal cell cycle. For HepG2 cells, this time was set to 48 h; which doubled the time period necessary for this type of cell (2 normal cycles). Concentrations were maintained as per those detailed in Section 4.5 and were also used in the cell cycle assay. Previous reports [18,45,46,47,48] and manufacturer’s instructions (Litron Laboratories, 2009) were followed, all according to the framework of the OECD Guideline [16].

Briefly, in 24-well plates, HepG2 cells (2 × 10^6^ cells/mL) were seeded and treated with BEA and OTA as detailed in Section 4.5 for 48 h. The day of the experiment Nucleic Acid Dye A Solution was added to the cells, placed on ice, and exposed to light for 30 min for photoactivation of the EMA flurorochrome dye. Afterwards, cells were washed and lysed with the Litron Lysis kit solution and preserved from light for 60 min. Afterwards, cells were gently resuspended and a lysis solution 2 containing Nucleic Acid Dye B (SYTOX flurorochrome) was added, incubated for 30 min at room temperature in the dark, and analyzed by flow cytometry. Gating cell population and analysis strategies were performed with the FACS Fortesa 7.1 software following the instructions and template provided with the in vitro microflow kit manual and as described by Bryce et al. [46,47]. The control cells received equal volume of the vehicle (cell culture medium). Etoposide was used as the positive control [45,47,49]. The percentage of micronucleus was determined after acquiring a total of 20,000 gated nuclei events per sample as indicated in OECD TG 487 for MN evaluation through flow cytometry.

### 4.8. Statistical Analysis

Statistical analysis of data was carried out using the IBM SPSS Statistic version 24.0 (SPSS, Chicago, IL, USA, 2017) statistical software package. Data were expressed as the mean ± SEM of four independent experiments. The statistical analysis of the results was performed by student’s *t*-test for paired samples. Differences between groups were statistically analyzed using ANOVA followed by the Tukey HDS post-hoc test for multiple comparisons. *p* ≤ 0.05 was considered statistically significant.

## Figures and Tables

**Table 1:** The medium inhibitory concentration (IC_50_) of beauvericin (BEA) and Ochratoxin A (OTA) in HepG2 cells after 24, 48, and 72 h of exposure by MTT assay.

Mycotoxins	IC_50_ (µM)		
	24 h	48 h	72 h
OTA	75 ± 0.04	52.62 ± 0.06	36 ± 0.09
BEA	12.5 ± 0.04	7.01 ± 0.05	5.5 ± 0.07

**Table 2:** The parameters *Dm, m,* and *r* are the antilog of x-intercept, the slope, and the linear correlation of the median-effect plot, which signifies the shape of the dose–effect curve, the potency (IC_50_), and the conformity of the data to the mass action law, respectively [7,8]. *Dm* and *m* values are used for calculating the CI value (CI < 1, =1 and >1 indicates synergism (Syn), additive (Add), and antagonism (Ant) effects, respectively. IC_25_, IC_50_, IC_75_, and IC_90_ are the doses required to inhibit proliferation at 25, 50, 75, and 90%, respectively. CalcuSyn Software provide automatically these values.

Mycotoxin	Time (h)	*Dm* (µM)	*m*	*r*	CI Values							
					CI_25_		CI_50_		CI_75_		CI_90_	
BEA	24	19.12	2.51	0.907								
	48	13.13	2.94	0.920								
	72	8.19	2.37	0.967								
OTA	24	86.73	2.02	0.960								
	48	77.57	4.24	0.915								
	72	46.64	4.01	0.956								
BEA + OTA	24	6.95	2.15	0.636	1.17 ± 2.48	Add	1.17 ± 3.8	Add	1.17 ± 5.8	Add	1.17 ± 8.9	Add
	48	1.79	1.86	0.948	0.27 ± 0.08	Syn	0.37 ± 0.14	Syn	0.49 ± 0.24	Syn	0.65 ± 0.42	Add
	72	0.95	1.57	0.987	0.22 ± 0.04	Syn	0.32 ± 0.06	Syn	0.46 ± 0.10	Syn	0.66 ± 0.19	Syn
